# High serum alpha-fetoprotein and positive immunohistochemistry of alpha-fetoprotein are related to poor prognosis of gastric cancer with liver metastasis

**DOI:** 10.1038/s41598-024-54394-1

**Published:** 2024-02-14

**Authors:** Yuriko Takayama-Isagawa, Kengo Kanetaka, Shinichiro Kobayashi, Akira Yoneda, Shinichiro Ito, Susumu Eguchi

**Affiliations:** 1 Department of Pathology, Jichi Medical University Hospital, Shimotsuke, Japan; 2 Department of Surgery, Nagasaki University Graduate School of Biomedical Sciences, Sakamoto 1-7-1, Nagasaki, 8528501 Japan

**Keywords:** Cancer, Gastrointestinal cancer

## Abstract

Liver metastasis in gastric cancer is incurable. Alpha-fetoprotein-producing gastric cancer has a poor prognosis and is prone to liver metastasis. We investigated the association between preoperative serum alpha-fetoprotein levels, liver metastasis, and expression of primitive enterocyte phenotype markers. We reviewed the medical records of 401 patients with gastric cancer who underwent curative surgical resection and immunohistochemically evaluated the primitive phenotype markers. The preoperative serum alpha-fetoprotein levels were elevated and normal in 8 and 393 patients, respectively. Liver metastasis was more frequent in patients with higher preoperative alpha-fetoprotein levels. The 5-year postoperative recurrence-free survival and overall survival rates were significantly worse in patients with higher preoperative serum alpha-fetoprotein levels. Although alpha-fetoprotein and Glypican3 and Spalt-like transcription factor 4 tended to be stained with high preoperative serum alpha-fetoprotein levels, these markers were also positive in some patients with normal alpha-fetoprotein levels. In summary, patients with gastric cancer and high preoperative serum alpha-fetoprotein levels have a poor prognosis and high incidence of liver metastasis. Alpha-fetoprotein can help detect liver metastasis relating to the primitive enterocyte phenotype.

## Introduction

Gastric cancer is the third leading cause of death due to cancer, globally^1^. The incidence and mortality of gastric cancer have been decreasing due to the eradication of pathogenic H.pylori, early diagnosis, and advancement of treatment via endoscopy. However, many patients could still develop distant metastasis, which is one of the non-curative factors. Liver metastasis accounts for 48% of gastric cancer metastasis^2^.

Over the past 3 decades, the treatment of hematological metastasis from colorectal cancer has improved, and local resection can now achieve a better prognosis. However, liver metastasis from gastric cancer is rarely considered a candidate for local resection because of the presence of extrahepatic metastasis such as peritoneal dissemination.

Recently, various chemotherapies have been used as definitive therapy for unresectable gastric cancer. Moreover, improvements in the prognosis of gastric cancer with liver metastasis through a combination of chemotherapy with an immune checkpoint inhibitor and hepatectomy are reported^3–5^. In this situation, it is important to select patients with a high risk of liver metastasis for early detection in their clinical course.

Various tumor markers, such as CEA and CA19-9, have been used for this purpose; however, the appropriate indications for using these markers in detecting liver metastasis remain unclear^6^.

Althoughα-fetoprotein (AFP) was originally considered a useful tumor marker for patient screening or monitoring for hepatocellular carcinoma (HCC) and yolk sac tumor^7,8^, it is known that some malignancies such as colorectal and lung cancer can produce also AFP^9–12^. AFP-producing gastric cancer has poor prognosis and is prone to liver metastasis^13^. In fact, various reports have shown the efficacy of preoperative examination of AFP values in predicting liver metastasis after curative gastrectomy^14–22^.

Recently, AFP positivity has also been associated with the expression of primitive phenotypic markers, such as glypican3 (GPC3) and Spalt like transcription factor 4 (SALL4). Wang et al. have defined this subtype as gastric adenocarcinoma with primitive enterocyte phenotype (GAPEP)^23^. Patients with GAPEP also have a poor prognosis and frequently exhibit distant metastasis^21,23^. Previous reports often focused on GAPEP phenotype expression in preoperative serum AFP-positive cases; however, the cutoff value is not uniform across the studies and it is possible that the GAPEP phenotype could contribute to the development of liver metastasis in “AFP-negative” gastric cancer also.

In this study, we investigated the relationship between preoperative serum AFP levels and clinicopathological features, including the development of metachronous liver metastasis after curative gastrectomy. Furthermore, to focus on the relationship between liver metastasis and GAPEP phenotype, we immunohistochemically evaluated the expression of AFP, GPC3, and SALL4 in gastric cancer with metachronous liver metastasis regardless of preoperative serum AFP levels.

## Results

The preoperative serum AFP levels in this study ranged from 0.6 to 44,613 ng/ml, with a median value of 3.1 ng/ml. Overall, eight and 393 patients with high (AFP-H) and low (AFP-N) preoperative serum AFP levels, respectively, were identified.

A comparison of the clinicopathological characteristics of the AFP-H and AFP-N groups is presented in Table 1. No significant associations with age, sex, or tumor location were observed. No difference was observed between the AFP-H and AFP-N groups in terms of lymphatic invasion, and histopathological grading; however, significant differences in depth of invasion, N status, vessel invasion, and stage were identified. Moreover, distant metastasis developed in 46% of the patients in the AFP-H group and in 13% of the patients in the AFP-N group.

**Table 1:** Association between clinicopathological features and preoperative serum AFP.

	AFP-H (n = 8)	AFP-N (n = 393)	*P* value
Age	77 (55–85)	70 (34–89)	0.147
Sex			0.732
Male/female	6/2	273/120	
Tumor location			0.217
Upper third	4	109	
Middle third	1	152	
Lower third	3	132	
Tumor size [mm]	67.5 (40–100)	35 (5–200)	0.0015
Depth of invasion			0.0066
T1	0	228	
T2	2	54	
T3	3	70	
T4	3	41	
N status			0.0079
N0	2	280	
N1	3	38	
N2-3	3	75	
Lauren classification			0.747
Intestinal/diffuse	4/4	219/174	
Lymphatic invasion			0.098
No/yes	2/6	214/179	
Vessel invasion			0.0004
No/Yes	0/8	216/177	
Stage			0.0006
I/II/III	0/4/4	258/60/75	
Distant metastasis	6 (62.5%)	50 (12.7%)	< 0.0001

There is a significant difference in distant metastasis between the two groups.

We further compared recurrence patterns between patients in the AFP-H and AFP-N groups (Table 2). Within 3 years after curative gastrectomy, 24 metachronous liver metastases and 24 peritoneal recurrences were encountered in all analyzed patients, and liver metastasis was most prevalent in patients in the AFP-H group. In contrast, elevated CEA and CA19-9 levels were significantly correlated with peritoneal recurrence and not with liver metastasis, indicating that the preoperative serum AFP level is a specific predictive marker for liver metastasis.

**Table 2:** Comparison of recurrence patterns.

Tumor marker	Liver*			Peritoneum*		
	Positive	Negative	*P* value	positive	Negative	*P* value
AFP-N	20	373	< 0.0001	24	369	0.471
AFP-H	4	4		0	8	
CEA < 5	18	325	0.130	15	328	0.0009
CEA 5 = <	6	52		9	49	
CA19-9 < 37	21	354	0.217	20	355	0.037
CA19-9 37 = <	3	23		4	22	

AFP was significantly correlated with liver metastasis, while CEA and CA19-9 was significantly correlated with peritoneal dissemination.

*Duplicates in the same case.

**Figure 1:**
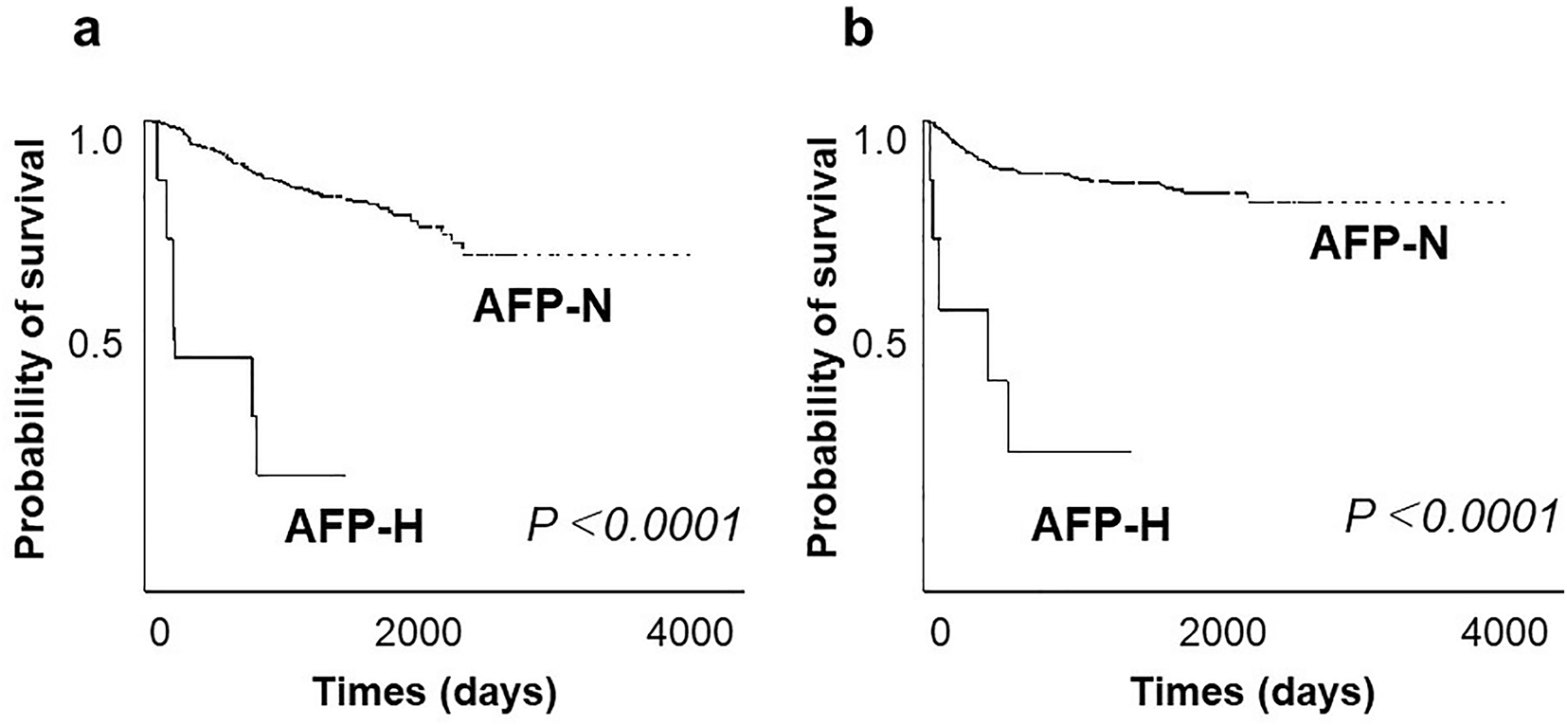
(**a**) Overall survival (OS) of all patients, (**b**) Recurrence-free survival (RFS) of all patients. The AFP-H group had a worse prognosis than the AFP-N group.

The postoperative 5-year RFS and OS rates of patients in the AFP-H group were significantly poorer than those in the AFP-N group (Fig. 1).

For all patients, univariate analysis showed that lymphatic and vessel invasion, preoperative serum AFP, CEA, CA19-9, and Stage were prognostic factors for both RFS and OS (Table 3 and Supplementary Table 1). In addition, multivariate analysis using Cox proportional hazards demonstrated that, in addition to tumor stage and CEA, preoperative serum AFP levels were independent prognostic factors for RFS.

**Table 3:** Prognostic factors for recurrence-free survival in 8 AFP-H patients.

	Univariate		Multivariate	
	HR (95% CI)	*P* value	HR (95% CI)	*P* value
Lauren classification				
Diffuse	Ref			
Intestinal	1.130 (0.668–1.912)	0.6494	–	
Lymphatic invasion				
No	Ref		Ref	
Yes	5.805 (3.006–11.21)	< 0.0001	0.846 (0.368–1.944)	0.6931
Vessel invasion				
No	Ref		Ref	
Yes	7.309 (3.585–14.90)	< 0.0001	1.976 (0.855–4.566)	0.1109
AFP*				
AFP-N	Ref		Ref	
AFP-H	9.816 (3.885–24.80)	< 0.0001	3.693 (1.410–9.674)	0.0078
CEA				
< 5	Ref		Ref	
5 = <	2.860 (1.620–5.049)	0.0003	1.295 (0.720–2.329)	0.3878
CA19-9				
< 37	Ref		Ref	
37 = <	3.743 (1.889–7.416)	0.0002	2.410 (1.185–4.904)	0.0152
Stage				
I	Ref		Ref	
II	11.67 (4.563–30.09)	< 0.0001	7.628 (2.589–22.40)	0.0002
III	27.85 (11.72–66.20)	< 0.0001	17.92 (6.151–52.23)	< 0.0001

Univariate and multivariate analyses revealed that preoperative serum AFP levels were significantly associated with OS and RFS.

*Preoperative serum AFP.

**Figure 2:**
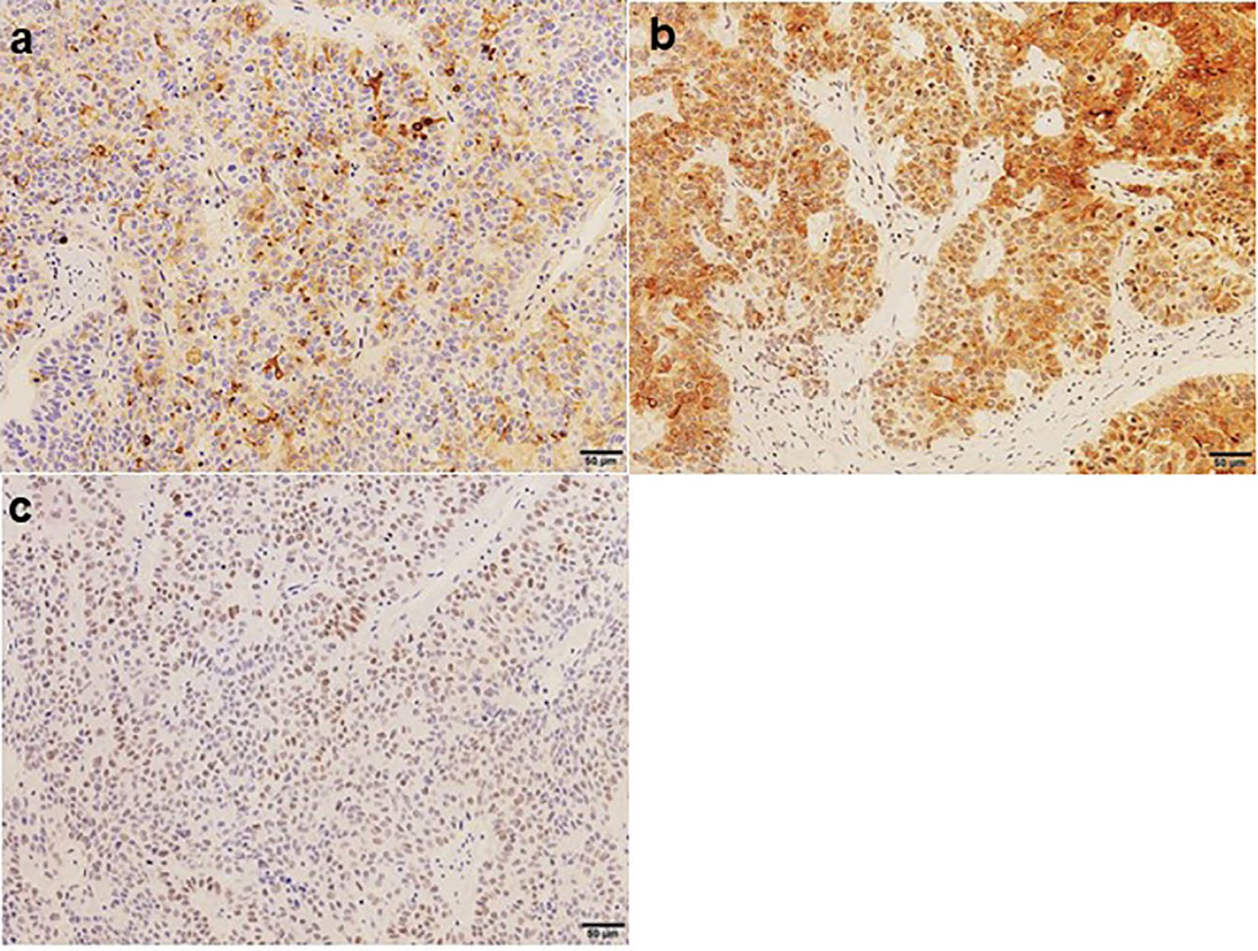
Representative examples showing positive staining for AFP (**a**) and GPC3 (**b**) in the cytoplasm and SALL4 (**c**) in the nucleus. This case belonged to the AFP-H group.

Immunohistochemistry was performed on 24 patient samples, which included 8 patients in the AFP-H group and 20 patients who developed metachronous liver metastasis in the AFP-N group. The results of the immunohistochemical analyses are summarized in Table 4. Within the AFP-H group, immunohistochemical staining showed that 7 cases were positive; the immunohistochemical positive ratio was 87.5% and all AFP-positive specimens were positive for both GPC3 and SALL4 (Fig. 2).

**Table 4:** Clinicopathological characteristics of 8 cases in AFP-H group.

Age	Sex	AFP* (ng/ml)	Size (mm)	pT	pN	ly	v	Histology	IHC**		
									AFP	GPC3	SALL4
74	F	44,613	100	2	0	1	1	tub2	+	+	+
80	M	368.8	65	2	2	1	1	por1	+	+	+
68	M	254.1	60	2	0	1	1	tub2	+	+	+
81	M	172.1	78	2	1	0	3	tub2	+	+	+
84	M	49.5	90	3	2	1	1	tub2	+	+	+
85	M	28.1	70	2	1	0	2	por1	+	+	+
68	M	20.7	53	1	0	1	2	tub1	+	+	+
55	F	20.7	40	1	0	3	2	por2	−	−	+

A positive status was observed when cytoplasmic staining for AFP and GPC3 was observed in 1% or more, and nuclear staining for SALL4 was observed in 10% or more.

Samples from patients with AFP-H group showed immunohistochemical features of GAPEP, and all AFP-positive specimens were immunohistochemically positive for both GPC3 and SALL4.

*Preoperative serum AFP.

**Immunohistochemical staining.

The expression in primary gastric cancer with metachronous liver metastasis, regardless of perioperative serum AFP levels, was analyzed (Table 5). Among these patients, we found five cases immunohistochemically positive for AFP. Nine cases were positive for GPC3 and seven cases were positive for SALL4. Thus, 41.7% of gastric cancer cases with metachronous liver metastases have the GAPEP phenotype. We also detected that even in the AFP-negative cases, 30% of patients with gastric cancer with metachronous liver metastasis had the GAPEP phenotype.

**Table 5:** Clinicopathological characteristics of all cases with metachronous liver metastasis.

Age	Gender	AFP* [ng/ml]	Size [mm]	pT	pN	ly	v	Histology	IHC**		
									AFP	GPC3	SALL4
74	F	44,613	100	2	0	1	1	tub2	+	+	+
68	M	254.1	60	2	0	1	1	tub2	+	+	+
84	M	49.5	90	3	2	1	1	tub2	+	+	+
68	M	20.7	53	1	0	1	2	tub1	+	+	+
67	M	7.9	45	2	1	1	2	tub1	+	+	+
82	M	3.6	55	4	3	2	2	tub2	−	−	−
81	M	1.5	48	3	1	2	2	tub1	−	+	+
77	F	2.5	30	3	0	3	3	tub2	−	−	−
66	F	1.7	55	3	0	0	2	tub1	−	−	−
69	M	4.9	90	3	0	1	3	tub2	−	−	−
69	M	3.2	79	3	0	0	3	tub2	−	−	+
73	M	3.1	50	3	1	1	3	por1	−	−	−
64	M	3.9	31	3	1	1	2	tub1	−	−	−
67	M	3.1	75	3	2	2	2	tub2	−	+	−
52	M	3.4	32	3	3	3	2	tub2	−	−	−
66	M	3.3	30	3	1	1	3	tub2	−	−	−
72	M	1.7	75	3	3	3	3	por1	−	+	−
78	M	2.1	25	1b	0	0	0	tub1	−	−	−
64	M	2.6	100	4b	1	3	2	tub2	−	−	−
76	M	1.1	150	4a	2	0	3	por1	−	−	−
73	F	4.7	40	4a	3	2	2	tub2	−	−	−
76	M	1.6	30	2	0	1c	1c	tub2	−	−	−
65	M	5.4	7	2	3a	1	3	por1	−	+	−
86	M	3.2	51	1a	0	0	0	tub1	−	−	−

Among the patients in the AFP-N group, immunohistochemistry was positive for AFP in one case, GPC3 in five cases, and SALL4 in three cases.

*Preoperative serum AFP, **Immunohistochemical staining.

Serum AFP was re-examined in 6 patients in the AFP-H group after surgery. The AFP levels fell in the normal range in three patients; however, three patients with persistently elevated postoperative AFP levels developed liver metastasis in the early postoperative period. One patient who was immunohistochemically positive for AFP in the AFP-N group showed an elevated serum AFP level of 28,807 ng/ml 9 months after the operation, and abdominal CT showed multiple liver metastases.

## Discussion

The present study showed that a high preoperative serum AFP level is an indicator of poor prognosis after curative gastrectomy. The study also showed that even in the AFP-negative cases, 30% of patients with gastric cancer with metachronous liver metastasis had the GAPEP phenotype. This finding implied that the GAPEP phenotype can universally contribute to the development of liver metastasis after gastrectomy.

Our report also indicated that different elevated markers might indicate the risk of different sites of recurrence. CEA and CA19-9 are routinely used as diagnostic and follow-up markers for gastric cancer. Our study revealed that preoperative elevation of these markers was a risk factor for peritoneal recurrence but not for liver metastasis. In contrast, elevated AFP was specifically associated with metachronous liver metastasis in our cohort.

There are many reports indicating the efficacy of AFP in predicting poor prognosis^14–22^. Most gastric cancers with high serum AFP levels exhibit a high incidence of lymphatic invasion, venous invasion, and liver metastasis. Adachi et al. reported a higher incidence of liver metastasis in addition to lymph node metastasis in the largest AFP-positive case series of 270 patients^24^. In the present study, we elucidated significant differences in the depth of tumor invasion, vessel invasion, lymph node metastasis, and distant metastasis between the AFP-positive and AFP-negative cases. In contrast, He et al. showed no correlation between serum AFP levels and lymph node metastasis or liver metastasis even in a larger cohort than ours^18^. This might be due to differences in the definition of AFP positivity such as the cutoff value of serum AFP or immunohistochemical staining for AFP.

The cutoff value of preoperative serum AFP level differs among studies^14,15,19,22,25^. We initially used AFP > 10 ng/ml as the cutoff value^26^, and among serologically positive gastric cancer cases, only seven cases were immunohistochemically AFP-positive (Supplementary Table 2). Our results revealed that most immunohistochemically positive AFP cases had a preoperative serum AFP level of > 20 ng/ml. To select immunoreactive cases, a cutoff value of 20 ng/ml seems reasonable. As a result, we finally used the cutoff value of 20 ng/ml in the analysis of clinicopathological factors; however, even if AFP positivity might be defined as preoperative serum AFP > 100 ng/ml, Wang et al. reported that only 29 cases showed immunohistochemical positivity among 45 serological AFP-positive patients^16^. Previous studies have also shown that in patients with high serum AFP, the positivity rate of immunohistochemistry for AFP is 64.5–93.7%^27^. Therefore, the correct cutoff value for AFP remains to be determined in future analysis.

In immunohistochemical analysis, all AFP-positive cases also showed positivity for the primitive phenotypic markers GPC3 and SALL4, which are reported to be more sensitive markers for fetal gut differentiation than AFP^21,22,28^. Wang et al. also reported a strong correlation between elevated serum AFP levels and immunohistochemical positivity for GAPEP markers^21^. However, it remains unclear to what extent the GAPEP phenotype might influence the occurrence of liver metastasis following radical gastrectomy. Our analysis revealed that 42% of gastric cancer cases that developed metachronous liver metastasis exhibited the GAPEP phenotype, irrespective of serum AFP elevation. This percentage included 30% of cases in the AFP-N group. This implied that the GAPEP phenotype can universally contribute to the development of liver metastasis after gastrectomy.

The importance of these primitive phenotypic markers in association with the highly aggressive nature of gastric cancer has been previously emphasized. Wang et al. reported that patients with the GAPEP phenotype have a poor prognosis and frequently exhibit liver metastasis^21,23^.

Although the molecular mechanism of the GAPEP phenotype in relation to a poor prognosis remains unknown, some reports have indicated differences in the molecular aspects of gastric cancer with and without the GAPEP phenotype. Maruyama et al. who performed a comprehensive micro RNA array-based study in AFP-positive patients, assumed that AFP-positive gastric cancer is completely different from AFP-negative gastric cancer, and that the mechanism of liver metastasis between the two is also distinct^29^. Amemiya et al. found that c-Met is over-expressed in AFP-positive patients and may be involved in cell growth and migration^30,31^. Kamei et al. reported that the expression of vascular endothelial growth factor was much higher in AFP-positive cases than in AFP-negative cases^32^. These differences may contribute to the characteristics of stronger proliferation, lower cell apoptosis and greater neovascularization in AFP-positive cancer than in AFP-negative gastric cancer^33^. GPC3 is expressed in some embryonic tissues, such as liver, and is involved in cell migration, proliferation, and survival in several tissues^34^. Shirakawa et al. also reported that GPC3 expression predicts a high risk of intrahepatic HCC recurrence^35^. SALL4 has been reported as a marker for a progenitor subclass of HCC with an aggressive phenotype^36,37^. Zeng et al. reported that SALL4 may regulate HCC stemness in a highly tumorigenic and invasive manner^38^.

Hirajima et al. emphasized that establishment of treatment strategy for liver metastasis due to gastric cancer like that of colorectal cancer is important to improve prognosis of AFP producing gastric cancer^14^.

They also reported that AFP production is not an independent prognostic factor for gastric cancer; rather, there is no effective treatment for liver metastasis from gastric cancer. They also reported that AFP-producing gastric cancer without liver metastasis did not necessarily have a poor prognosis.

Therefore, the development of effective treatments for liver metastasis could improve the prognosis of AFP-producing gastric cancer^39–41^. Chen et al. reported that perioperative chemotherapy combined with surgery could improve the prognosis of gastric cancer with liver metastasis^42^. Li et al. also indicated that adjuvant therapies after surgery were most likely to result in better treatment of gastric cancer with liver metastasis^43^. However, there is still no standard chemotherapy regimen for patients with gastric cancer and liver metastasis worldwide.

Simmet et al. revealed that a cisplatin-based regimen achieved a partial or complete response in 11 patients with metastatic hepatoid adenocarcinoma^44^. Kamiimabeppu et al. showed that ramucirumab-containing chemotherapy resulted in a higher response and disease control rate in an AFP-positive gastric cancer group than in an AFP-negative group^45^. The authors concluded that AFP-positive gastric cancer might benefit more from this regimen. Li et al. also indicated the possible benefit of immune checkpoint inhibitors combined with chemotherapy for the treatment of AFP-positive gastric cancer^46^.

A recent meta-analysis indicated that patients with a single liver metastasis can achieve long-term survival with local treatment such as surgical resection and radiofrequency therapy if indication could be appropriated^5,43,47,48^ The Guidelines Committee of the Japanese Gastric Cancer Association also reconsidered local treatment as a beneficial strategy for patients with liver metastasis^49^.

This study has several limitations. First, this was a retrospective study; therefore, the possibility of benign etiologies cannot be eliminated. Second, the number of patients included in this study was small and we could not exclude the influence of the skewed sample size on the study outcome and statistical analysis. Third, although most patients in stages II and III might have been administered adjuvant chemotherapy with tegafur gimeracil oteracil potassium (S1) according to the Japanese guideline for gastric cancer in the analyzed period^50^, we did not analyze postoperative chemotherapy.

In conclusion, our result showed that gastric cancer with high serum AFP level was associated with a poor prognosis and a high incidence of liver metastasis.

Furthermore, we showed that even in the AFP-negative cases, 30% of gastric cancer cases with metachronous liver metastasis had the GAPEP phenotype. In a situation where curative treatment for liver metastasis is becoming available, preoperative prediction and early detection of liver metastasis after operation are critical. Because serum AFP may be the only available marker for gastric cancer cases with the GAPEP phenotype, perioperative monitoring of serum AFP level is useful for the early detection of liver metastasis relating to the GAPEP phenotype.

## Material and methods

### Patients and data

We included 401 patients with primary gastric cancer who underwent curative resection between January 2009 and December 2019 at Nagasaki University Hospital. Patients with chronic hepatitis, liver cirrhosis, or HCC were excluded based on the imaging and blood chemistry results. Peripheral blood samples were obtained upon initial presentation to the hospital.

Resected specimens were examined by pathologists based on the 15th Japanese classification of Gastric Carcinomas (JCGC) guidelines. Histological types were also classified as intestinal (papillary, moderately, and well-differentiated adenocarcinoma) and diffuse (poorly, signet-ring cell carcinoma and mucinous adenocarcinoma) types based on the Lauren classification.

At first, the diagnostic value of serum CEA, CA19-9, and AFP was compared. The cutoff values for CEA and CA19-9 were 5 ng/ml and 37 ng/ml, respectively. As there was no uniform cutoff value for AFP, we selected a value of 20 mg/dl according to previous reports. We categorized patients into two groups, namely AFP-H and AFP-N, using the designated cutoff value, and subsequently assessed the correlation between serum AFP levels and clinicopathological factors. Medical records were reviewed retrospectively, and the clinicopathological characteristics including age, sex, preoperative serum AFP level, tumor size, tumor location, histological differentiation, depth of invasion, peripheral lymph node invasion, clinical stage, and metachronous distant metastasis were obtained.

The follow-up program schedule for all patients included regular physical examinations and blood tests. Computed tomography was performed annually in patients with stage I tumors and every 6 months in patients with stage II or higher tumors for the first 5 years. Postoperative recurrence was diagnosed based on patient interviews, physical examination, and imaging results.

Follow-up information included the location of the postoperative recurrence site.

We defined “liver metastasis” as the development of liver metastasis within 3 years after curative gastrectomy; synchronous liver metastasis was excluded. This study was approved by the Ethics Committee of Nagasaki University Graduate School of Biomedical Sciences.

### Immunohistochemistry

Resected specimens were fixed in a 10% neutral buffered formaldehyde solution and embedded in paraffin. They were then stained with hematoxylin and eosin and examined histologically. Immunohistochemical staining was performed using primary antibodies against AFP (1:100, IR500, Dako), GPC3 (1:100, ab219313, Abcam), and SALL4 (1:300, ab181087, Abcam). The results were considered positive when > 1% cytoplasmic staining for AFP and GPC3 and > 10% nuclear staining for SALL4 were observed.

### Statistical analyses

Statistical analyses were conducted using SPSS (version 15.0 for Windows; SPPS Inc., Chicago, IL, United States). The χ^2^ test and the Mann–Whitney U test were used to assess the significant differences in the clinicopathological characteristics. Overall survival (OS) was measured from the time of resection until death or the last follow-up. Recurrence-free survival (RFS) was measured from the time of resection until the recurrence of gastric cancer. OS and RFS were analyzed using Kaplan–Meier curves and compared using the log-rank test. All significant variables observed in univariate analysis were included in the multivariate logistic regression analysis. Significance was set at *P* < 0.05.

### Human rights statement and informed consent

All procedures followed were in accordance with the ethical standards of the responsible committee on human experimentation (institutional and national) and with the Helsinki Declaration of 1964 and later versions. Informed consent to be included in the study, or the equivalent, was obtained from all patients.

### Supplementary Information

Supplementary Tables.

## Data Availability

All data generated or analyzed during this study are included in this published article.
