# Supplementary material for: High serum alpha-fetoprotein and positive immunohistochemistry of alpha-fetoprotein are related to poor prognosis of gastric cancer with liver metastasis
Source: Sci Rep. 2024 Feb 14;14:3695. doi: 10.1038/s41598-024-54394-1 (PMC10866906; doi:10.1038/s41598-024-54394-1)
Supplement: Supplementary file 1 — Supplementary Tables. [file 41598_2024_54394_MOESM1_ESM.pdf]

Supplementary Table 1. Prognostic factors for overall survival in 8 AFP-H patients

	Univariate		Multivariate	
	HR (95% CI)	P value	HR (95% CI)	P value
Lauren classification				
Diffuse	ref	0.816		
Intestinal	1.056(0.667-1.670)			
Lymphatic invation				
No	ref	<0.0001	ref	0.2061
Yes	2.686(1.658-4.349)		1.538(0.789-2.298)	
Vessel invasion				
No	ref	<0.0001	ref	0.0029
Yes	4.072(2.394-6.928)		2.143(1.111-4.133)	
AFP*				
AFP-N	ref	0.0002	ref	0.0024
AFP-H	5.88 (2.80-12.3)		3.928(1.621-9.516)	
CEA				
<5	ref	0.0012	ref	0.1955
5=<	2.386(1.412-4.030)		1.434(0.831-2.476)	
CA19-9				
<37	ref	0.0178	ref	0.1922
37=<	2.328(1.158-4.684)		1.611(0.787-3.300)	
Stage				
I	ref		ref	
II	4.347 (2.355-8.025)	<0.0001	3.198(1.524-6.712)	0.0021
III	6.900 (3.987-11.94)	<0.0001	5.160(2.414-11.03)	<0.0001

*preoperative serum AFP

Supplementary Table 2. Clinicopathological characteristics of cases in AFP-H group

Age	Sex	AFP* (ng/ml)	Size (mm)	pT	pN	ly	v	Histology	IHC** AFP	IHC** GPC3	IHC** SALL4
74	F	44613	100	2	0	1	1	tub2	+	+	+
80	M	368.8	65	2	2	1	1	por1	+	+	+
68	M	254.1	60	2	0	1	1	tub2	+	+	+
81	M	172.1	78	2	1	0	3	tub2	+	+	+
84	M	49.5	90	3	2	1	1	tub2	+	+	+
85	M	28.1	70	2	1	0	2	por1	+	+	+
68	M	20.7	53	1	0	1	2	tub1	+	+	+
55	F	20.7	40	1	0	3	2	por2	-	-	+
61	F	14.4	60	1	0	1	0	por2	-	-	-
77	M	12.9	20	1	0	0	0	tub1	-	-	-
70	M	11.1	20	1	0	1	0	por1	-	-	-
83	M	10.2	62	1	0	0	0	tub2	-	-	-
52	M	10.2	12	1	0	0	0	sig	-	-	-

*preoperative serum AFP

**Immunohistochemical staining
